# Engaging, recruiting, and retaining pregnant people from marginalized communities in environmental health cohort studies: a scoping review

**DOI:** 10.1186/s12889-025-22033-7

**Published:** 2025-03-07

**Authors:** Ghazal S. Fazli, Erica Phipps, Eric Crighton, Anglena Sarwar, Jillian Ashley-Martin

**Affiliations:** 1 Department of Geography, Geomatics, and the Environment, University of Toronto Mississauga, Mississauga, ON Canada; 2 Department of Health and Society, University of Toronto Scarborough, Toronto, ON Canada; 3 Department of Geography, Environment and Geomatics, Prenatal Environmental Health Education (PEHE) Collaboration, University of Ottawa, Ottawa, ON Canada; 4 Canadian Partnership for Children’s Health and Environment, Ottawa, ON Canada; 5 Environmental Health Science and Research Bureau, Health Canada, Ottawa, ON Canada

**Keywords:** Cohort, Pregnancy, Marginalized, Recruitment, Engagement, Retention, Environmental chemicals

## Abstract

**Objectives:**

To identify barriers to and strategies for improving the representation of pregnant people from marginalized communities in pregnancy cohort studies that measure environmental chemicals.

**Methods:**

Guided by the Arksey O’Malley and Levac Frameworks, we conducted a scoping review of peer-reviewed literature published between 2000 and 2022. Included studies discussed barriers and/or strategies related to engaging, recruiting, and retaining pregnant participants or participants of reproductive age from marginalized communities into environmental health research.

**Results:**

Twenty-nine peer-reviewed articles were included in the review. Overall, 31% (9/29) of the studies reported on engagement, recruitment, and retention of participants from racialized communities, 10% (3/29) reported on involvement of participants identifying as Indigenous, and 10% (3/29) of studies reported on participants living in households or areas of low socioeconomic status. We identified four key barriers: participant burden, social inequities, lack of trust, and lack of cultural relevance. We reported identified strategies to mitigate these barriers.

**Conclusion:**

Although there is limited coverage in the literature on strategies to effectively engage people from marginalized communities in environmental health pregnancy cohort studies, our findings suggest that applying a health equity and social justice lens to research may help address barriers that exist at the individual, interpersonal, community, institutional, and policy levels. Findings from this review may have important implications for planning future pregnancy cohort studies and ensuring that communities who are disproportionately affected by environmental chemical exposures may be better represented in research and considered in policy decisions.

**Supplementary Information:**

The online version contains supplementary material available at 10.1186/s12889-025-22033-7.

## Background

Environmental chemical exposures have important implications for health throughout the life course [1–19]. These chemicals include a wide range of substances that are naturally occurring (e.g., mercury, arsenic) as well as those produced in industrial settings and used in household and consumer products and in agriculture (e.g., phthalates, phenols, perfluoroalkyl substances (PFAS), pesticides). Many of these chemicals have demonstrated toxicity. For example, exposure to lead and mercury are associated with detrimental effects on children’s brain function that may persist throughout their life [20]. Exposure to PFAS may increase risk of pregnancy complications such as preeclampsia putting both the mother and child at risk of life-long health effects [21].

Although there are well-established associations between early life environmental chemical exposures and adverse effects on child health, not all populations experience equal burdens of exposure. In particular, people living in marginalizing circumstances, including those living on low income, Indigenous peoples, and racialized communities, are often disproportionately exposed to environmental chemicals [22, 23]. This disproportionate exposure may be driven by multiple potentially overlapping factors including residential proximity to chemical ‘hotspots’ (e.g., landfills, industry, high traffic zones), residence in older housing, or use of culturally relevant products containing high levels of chemicals (e.g., hair straighteners) [24]. Additionally, people living in marginalizing circumstances may be more susceptible to the adverse effects of environmental chemicals due to risk factors such as low nutritional status or higher levels of stress. For example, having limited access to healthcare and restricted financial resources may exacerbate underlying health conditions and the health effects of chronic stress [25]. While pregnancy cohort studies provide promising avenues to understand the relationships between environmental exposures and health outcomes [26], problematically, marginalized communities are often underrepresented in these studies [22].

Strategies typically used to recruit and retain pregnant participants in pregnancy cohorts may fail to account for the circumstances of marginalized populations [27–31]. Recruitment strategies are often tailored towards participants who have the resources to attend study visits during working hours and have access to transportation and child care [32]. Participants in existing cohorts and biomonitoring studies in middle to high income countries, for example, tend to be of moderate to high socioeconomic status and primarily represent non-racialized populations [33–39]. Consequently, these studies are not able to fully assess levels of exposures in marginalized communities or understand the factors that exacerbate exposure and susceptibility. Furthermore, human biomonitoring data, the cornerstone of exposure assessment in environmental epidemiology, require the collection and storage of human biological specimens for analysis of chemicals. This aspect of research may pose a particular barrier to recruitment as it is often invasive and requires a certain level of trust with the research team and process [31].

Strategies to improve representation of marginalized communities in pregnancy cohort studies are necessary. We conducted a scoping review to assess engagement, recruitment and retention strategies. In doing so, we contextualized the learnings from included studies by mapping our observations onto the socioecological model of health [40, 41]. This model is an effective tool for unpacking the multiple facets of marginalization and contextualizing how barriers to research participation can exist at individual, institutional, community, and policy levels [42–44].

Previous reviews have focused on the recruitment of pregnant people into clinical trials [45, 46] and pregnancy cohort studies [31]; however, no identified reviews have explored the issues specific to engaging, recruiting, and retaining marginalized communities in pregnancy cohort studies that collect biomonitoring data. In light of this gap, we conducted this review to inform recruitment strategies for forthcoming Canadian longitudinal biomonitoring research. Our primary objective was to identify barriers to and strategies for improving the representation of prospective parents and pregnant people from marginalized communities into pregnancy cohort studies that investigate associations between environmental chemicals and health outcomes. Our secondary objective was to map these barriers and strategies onto the socioecological model of health.

## Methods

We used the Arskey O’Malley framework and Levac’s further enhanced framework to conduct this review [47, 48]. These frameworks provide a robust approach for conducting scoping reviews and promote engagement with collaborators and stakeholders to elicit feedback related to the study protocol and interpretation of final study results. We report methodology and results according to the Preferred Reporting Items for Systematic Reviews and Meta-Analyses extensions for Scoping Reviews (PRISMA-ScR) (Suppl Table 1) [49].

### Search parameters

Relevant health research databases, namely, OVID Medline, OVID EMBASE, OVID APA, PsycINFO, Scopus, and EBSCO (CINAHL) were searched to identify peer-reviewed publications between January 1, 2000 – February 14, 2022. A library liaison was consulted on the search protocol and steps. The search was conducted first by identifying Medical Subject Headings (MeSH) terms followed by keywords (Table 1). The search was structured to identify literature in the following concepts: 1) marginalized, 2) engagement, recruitment, and retention, 3) environmental chemicals, and 4) cohort studies. Consistent with previous literature [42], we used the terms *engagement and recruitment* to refer to strategies for connecting with and enrolling participants into pregnancy cohort studies, and *retention* to refer to strategies for keeping participants engaged and motivated until the completion of the study.

**Table 1:** Search strategy

Concept	Search strategy
Marginalization	(Vulnerab* OR hard to reach OR seldom heard OR hidden group OR disadvantage* OR underrepresented OR under-represented OR underserved OR under-served OR low income OR low-income OR poor income OR low socioeconomic status OR low socioeconomic position OR low literacy OR low health literacy OR raciali#ed OR ethnic minorit* OR refugees OR asylum seekers OR deprived OR oppressed OR marginali* OR newcomer* OR immigrant* OR at risk OR at-risk OR minority OR minority health OR disabilit* OR disabled OR Indigenous OR Aboriginal OR First Nations OR Inuit OR Metis OR M#tis OR nonbinary OR LGBTQ OR LGBTQS OR LGBTQS2 OR LGBTQS2 + OR 2SLGBTQ +).tw,kf
Engagement/Recruitment/Retention	(Engag* OR patient engag* OR participant engag* OR public engag* OR patient outreach OR public outreach OR community member OR community participation OR patient participation OR public participation OR public involvement OR patient involvement OR patient selection OR public selection or recruitment OR participant recruitment OR retention* OR participant retention OR retentive*).tw,kf
Environmental chemicals	Biomonitor* OR monitor* OR biomarker OR exposure biomarker OR marker OR biological monitoring OR human biomonitoring OR human biomonitoring data OR human biomonitoring research OR environmental chemical* OR chemical* OR Lead OR arsenic OR mercury OR cadmium OR manganese OR phthalates OR bisphenol A OR BPA OR polybrominated diphenyl ethers OR PBDEs OR organophosphate OR OP OR pesticides OR polychlorinated biphenyls OR PCBs OR triclosan OR cotinine OR perfluoroalkyl substances OR PFASs OR metals OR parabens OR phenols OR Pesticides OR flame retardants).tw,kf
Cohort Studies	(Cohort OR cohort studies OR prospective* OR longitudinal* OR population based OR population based cohort or population based cohort stud*).tw,kf

This search strategy includes the Medical Subject Headings (MeSH) terms followed by keywords that were used to search relevant health research databases, namely, OVID Medline, OVID EMBASE, OVID APA, PsycINFO, Scopus and EBSCO (CINAHL) to identify peer-reviewed publications between January 1, 2000 – February 14, 2022, in consultation with a library liaison

### Study selection

After conducting the search and eliminating duplicates using Covidence review management software [50], one reviewer screened all identified articles by title and abstract, followed by a review of the full text and initial extraction of relevant data. A second reviewer also screened the included articles to ensure they met our inclusion criteria. This reviewer also conducted a thorough analysis and quality control check of all tables and text.

**Fig. 1:**
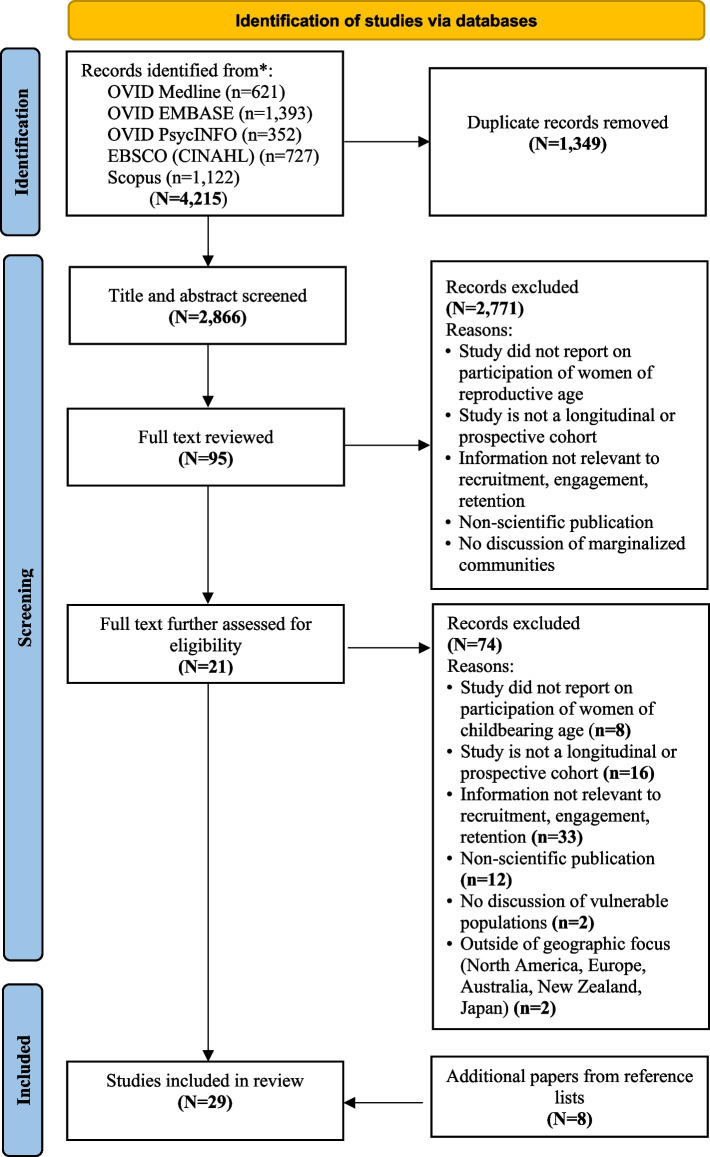
Preferred Reporting Items for Systematic Reviews and Meta-Analyses (PRISMA) Flowchart. Adapted from: Page MJ, McKenzie JE, Bossuyt PM, Boutron I, Hoffmann TC, Mulrow CD, et al. The PRISMA 2020 statement: an updated guideline for reporting systematic reviews. BMJ 2021;372:n71. doi: 10.1136/bmj.n71

Included publications were cohort studies that recruited people who were pregnant or of reproductive age, and provided information relevant to recruitment, engagement, or retention in marginalized populations. We focused on higher-income regions (e.g., North America, Europe, New Zealand, Japan, and Australia) as these were considered most relevant to our objective to inform potential future Canadian biomonitoring studies. We operated under the assumption that these study settings would have findings broadly generalizable to the capacity, resources, and funding in the Canadian setting. All included publications were peer-reviewed and written in English. As a final step, reference lists of publications selected for inclusion were scanned for any additional eligible studies (Fig. 1).

### Data extraction and analysis

**Fig. 2:**
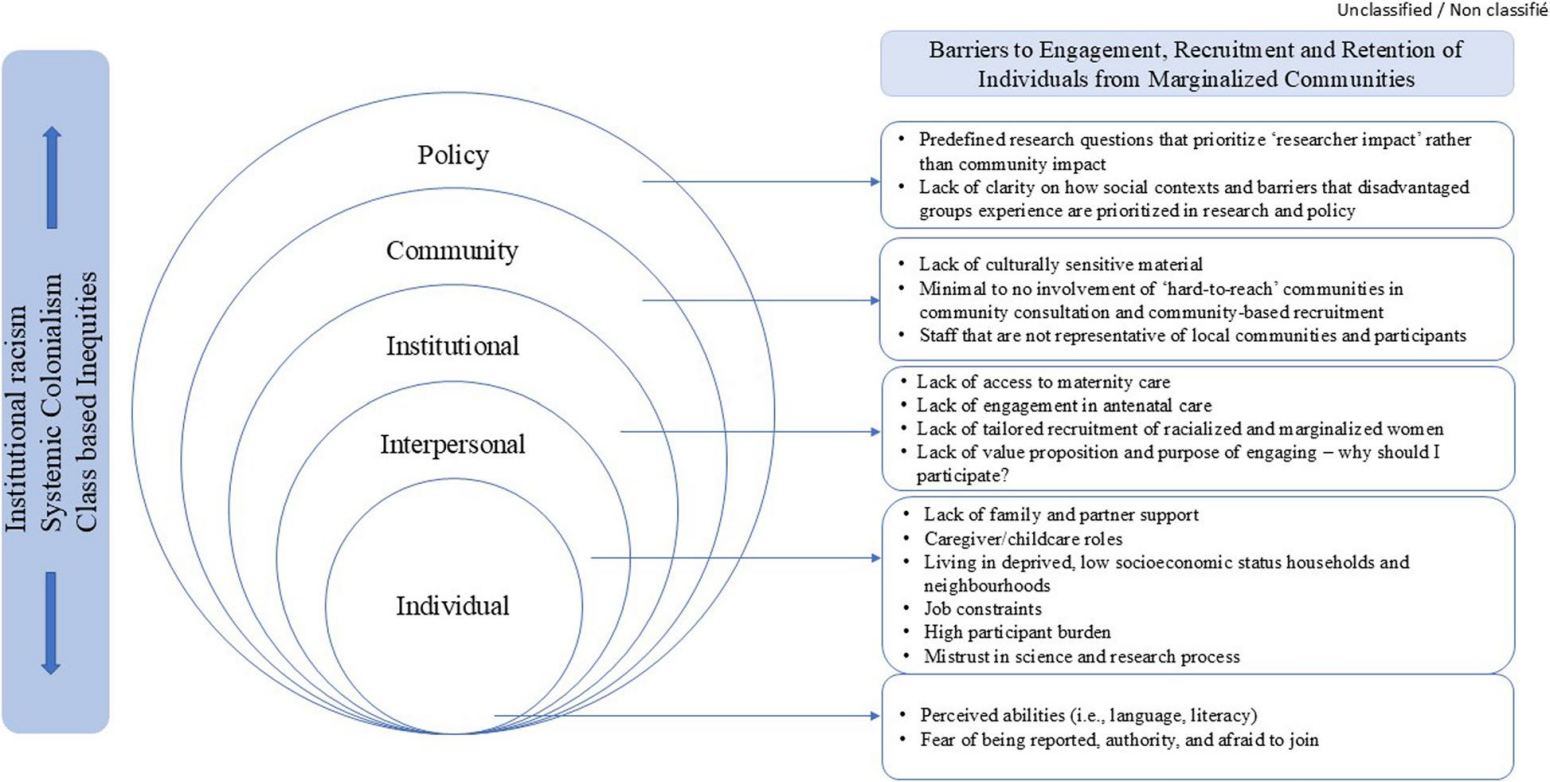
Mapping barriers to research participation among marginalized population. Adapted from: Socio-ecological model: framework for prevention, Centers for Disease Control. Available from: https://www.cdc.gov/violenceprevention/about/social-ecologicalmodel.html

The following data were extracted: author last name and date of online publication, study setting, name of cohort, sample size, attrition, number of participants retained at follow-up, and participant characteristics including age at time of recruitment, race, ethnicity, immigrant status, and socioeconomic status or income. We also retrieved information on recruitment setting, recruitment duration, and barriers and strategies to engagement, recruitment, and retention. One reviewer extracted data into a Microsoft Excel file prior to data synthesis. A thematic analysis was conducted using inductive and deductive approaches to identify themes corresponding to *barriers* that impede engagement, recruitment, and retention and *strategies* that promote successful engagement, recruitment, and retention of individuals from marginalized communities [51]. Themes were then mapped onto an adapted version of the socioecological model of health (Fig. 2).

### Consultation with stakeholders

Consistent with a scoping review approach that includes knowledge users [52, 53], we conducted two online consultation exercises with existing research collaborators and stakeholders from the Prenatal Environmental Health Education (PEHE) Collaboration and lead Health Canada scientists from the Maternal-Infant Research on Environmental Chemicals (MIREC) study. The purpose of this consultative process was to 1) review the search protocol and provide an overview of preliminary findings and 2) share the results of this review and discuss whether the results align with research and practice. We shared meeting notes with the PEHE-MIREC stakeholders and integrated their feedback into our global interpretation of the scoping review. A description of this knowledge users consultation and link to the full report is available on the PEHE website [54].

## Results

### Descriptive summary

The literature search yielded 2,866 articles, which were further screened by title and abstract according to the eligibility criteria, leaving 95 articles for full-text screening (Fig. 1). Of these, 21 articles met the study eligibility criteria. Reference lists were then scanned, yielding an additional eight articles. Altogether 29 articles were included for analysis. The majority of the studies were from the United States (8/29; 27%) and Canada (6/29; 21%), with the remainder from European countries (France, Germany, Belgium, Norway, Italy and Spain), New Zealand, Australia, and Japan. Thirty one percent (9/29) of the studies reported on engagement, recruitment, and retention of participants from racialized or marginalized communities, 10% (3/29) reported on involvement of participants identifying as Indigenous, and 10% (3/29) of studies reported on participants living in households or areas of low socioeconomic status. The most common recruitment setting was a medical clinic (62%, 18/29), followed by community settings (17%, 5/29), and a combination of clinic and community settings (14%, 4/29); online recruitment was the least common approach (10%, 3/29) (Table 2).

**Table 2:** Study characteristics of the included articles (*n* = 29)

Author and date	Country	Name of Cohort	Population	Sample Size	Attrition (loss to follow up)	Retained at follow-up	Age (provided in mean age, or age range of participants included (years)	Status, Race or Ethnicity or linguistically diverse	Socio-economic status/income
Ashman 2016 [55]	Australia	Gomeroi gaaynggal study	Pregnant people	236	110	66	13.8–40.9	Indigenous Australian	-
Bartholomew 2015a [37]	New Zealand	Growing Up in New Zealand	Pregnant people and their partner	6,822	-	6,012	20–40	Indigenous and non-Indigenous (European as reference group, Maori, Pacific, Asian and other)	Area income
Bartholomew 2015b [56]	New Zealand	Growing Up in New Zealand	Pregnant people and their partner	6,822	255	6,567	20–40	Indigenous and non-Indigenous (European, Maori, (Indigenous, Pacific, Asian and Other)	Area income
Bastain 2019 [57]	United States	Maternal And Developmental Risks from Environmental and Social Stressors (MADRES)	Pregnant people	523 (with plans to recruite 1000)	33	490	≥ 18	Hispanic (minority and low income)	Household income
Begum 2012 [58]	Canada	Alberta Pregnancy Outcomes and Nutrition (APrON) Study	Pregnant people and infants	600	29	571	≥ 16	-	-
Chasan-Taber 2009 [59]	United States	Proyecto Buena Salud	Pregnant people	1,626	76	1,308	16–40	Hispanic, African American, non-Hispanic White, and other	-
Ernst 2015 [60]	Germany	-	Pregnant people	48	-	-	22–45		
Faro 2021 [61]	United States	Child Health Out-comes (ECHO) Program	Pregnant people	47 ECHO Cohorts	-	-	-	-	-
Garcia-Blanco 2018 [62]	Spain	-	Pregnant people	243	-	-	-	-	-
Gracie 2010 [63]	Canada	All Our Babies Cohort Study	Pregnant peoplep	2,200	400	1,800	≥ 18	-	-
Hertz-Picciotto 2010 [64]	United States	SUPERB (Study of Use of Products and Exposure Related Behaviour)	Families (one parent and one child) and older individuals (age 55 +)	499 (parents), 566 (children), 156 (older adults)	-	-	-	-	-
Hertz-Picciotto 2018 [65]	United States	MARBLES (Markers of Autism Risk in Babies‚ Learning Early Signs)	Pregnant people	463	75	388	≥ 30	Hispanic, non-Hispanic Black, Asian, or multiracial	-
Kawamoto 2014 [66]	Japan	Japan Environment and Children Study (JECS)	Pregnant people	100,000	-	-	-	-	-
Lara-Cinisomo 2016 [67]	United States	NA	Pregnant people	34	5	29	-	Immigrant Latina peoples born in the U.S. or outside (Mexico, Guatemala, El Salvador)	
Loubet 2016 [68]	France	G-GrippeNet (Grossesse-GrippeNet in French,Pregnancy-GrippeNet)	Pregnant people	153	-	-	-	-	-
Loxton 2015 [38]	Australia	Australian Longitudinal Study on People’s Health (ALSWH) and People’s Health of Australia! (WHoA!)	Female	17,069	-	-	18–23	-	-
McDonald 2013 [86]	Canada	All Our Babies (AOB) study	Pregnant people	4,011	623	3,388	≥ 18		
Manca 2013 [69]	Canada	Alberta Pregnancy Outcomes and Nutrition (APrON)	Pregnant people	1,200	-	-	-	-	-
Morrens 2017 [81]	Belgium	Flemish Environment and Health Study	Pregnant people	281	-	-	-	People of Turkish and Moroccan descent	-
Morton 2014 [70]	New Zealand	Growing Up in New Zealand	Pregnant people and their partner	6,822	-	-	-	-	-
Postma 2016 [71]	United States	The National Children Study (NCS)	Hispanic people and their families	159	-	-	18–49	Hispanic people	-
Quante 2012 [72]	Germany	LIFE Child BIRTH Study	Pregnant people and their partners	2,000					
Richiardi 2007 [73]	Italy	NINFEA Cohort	Pregnant people	670				-	-
Smith 2021 [74]	United States	Food, Feelings, and Family (FFF) Study	Pregnant people	167	125	42	18–35	-	-
Spallek 2020 [75]	Germany	BaBi-Stress study (Bielefeld Germany) and BaBeK study (Berlin Germany)	Pregnant people	144	-	-	≥ 18	Immigrant of Turkish origin	-
von Ruesten 2014 [76]	Norway	Norwegian Mother and Child Cohort Study (MoBa)	Pregnant people, children, and fathers	Children (114,500), Mothers (95,200), Fathers (75,200)	-	-	-	-	-
Walker 2011 [77]	Canada	The Ottawa and Kingston (OaK) Birth Cohort	Pregnant people	8,085	-	-	30.4	-	-
Webster 2012 [78]	Canada	Chemicals, Health and Pregnancy study (CHirP)	Pregnant people	308	156	152	≥ 19	Predominantly white	-
Zook 2010 [79]	United States	Urban Environment and Childhood Asthma (URECA)	Pregnant people	606	67	539	13–42	Predominantly Hispanic and non-Hispanic Black	

### Barriers to engagement, recruitment, and retention in pregnancy cohort studies

**Fig. 3:**
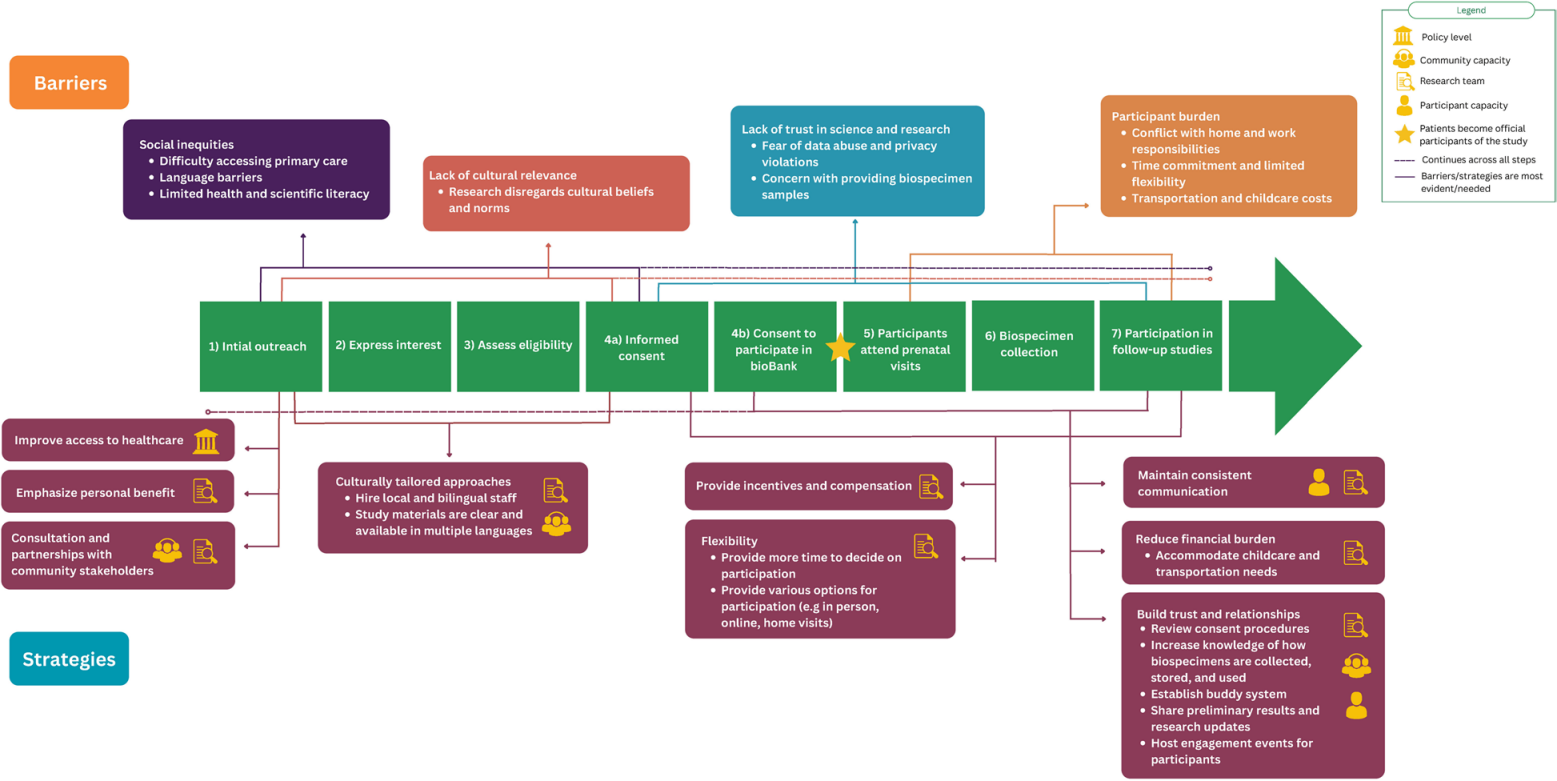
Barriers and strategies to engaging, recruiting and retaining pregnant people from marginalized communities in environmental health cohort studies

The primary barriers to engagement, recruitment, and retention are *participant burden, socioeconomic inequalities, lack of trust in science and research* and *lack of cultural relevance* (Table 3). To demonstrate the influence of individual, interpersonal, community, institutional, and policy factors and influences, we mapped barriers to the socioecological model (Fig. 2). To support the practical application of our findings, we also mapped identified barriers onto a timeline depicting a typical sequence of participant engagement, recruitment, and retention activities in a cohort study design (Fig. 3).

**Table 3:** Identified themes on barriers and strategies for recruitment, engagement, and retention

Author and date	Recruitment setting	Recruitment method	Recruitment duration	Barriers to engagement, recruitment, and retention	Strategies and approaches for engagement, recruitment, and retention (Effectiveness)
Ashman 2016 [55]	Clinical settings (Antenatal)	Pregnant people were recruited by Indigenous research assistants at one of two antenatal clinics (i.e., Indigenous antenatal birth services)	2010-present	**Engagement & Recruitment:** • *Time commitment is burdensome* for people **Retention:** • *Lack of continuity and irregularity* of attendance at antenatal visit	**Engagement & Recruitment:** • *Community consultation* – before the onset of the study, researchers engaged in a 2-year process of community consultation with Indigenous stakeholders, including Elders, mothers‚ schools, employment agencies and local Indigenous health organizations • *Hiring Indigenous staff* to share expertise on community connections and knowledge **Retention:** • *Building connection and trust* between staff and participants
Bartholomew 2015a [37]	Clinical settings (Antenatal)	People were recruited in antenatal clinics with Lead maternity carer (LMC)	2009–2010	**Engagement & Recruitment:** • *Timeliness of engagement* with partners involved (i.e., health care providers) and participants who are particularly in their **first pregnancy**, and people living in more **socioeconomically deprived area** • *Significant personal, health ****literacy-related barriers in disadvantaged group*** **Retention:** • *Address the persistent equity issues* in access to maternity care	**Engagement & Recruitment:** Not reported **Retention:** Not reported
Bartholomew 2015b [56]	Clinical settings (Antenatal)	People were recruited in antenatal clinics with LMC	2009–2010	**Engagement & Recruitment:** • ***Disparity in engaging marginalized**** and racialized people* where people not engaging a maternity care provider were more likely to be non-European, < 20 years or > 40 years old, with **poorer educational attainment**, or living in more deprived households **Retention:** Not reported	**Engagement & Recruitment:** • *Need for policies* to improve engagement in antenatal care **Retention:** Not reported
Bastain 2019 [57]	Community Health Clinics	People were recruited from four community health clinics	Nov 2015-Oct 2018	**Engagement & Recruitment:** NA **Retention:** NA	**Engagement & Recruitment:** • *Established relationships* with a lead clinic physician to engage and recruit participants • *Approach potentially eligible* people at each clinic site and conduct in-person and telephone interviews • *Advertisements placed in local papers* or in community locations indicating selected criteria for self-referral • **Retention:** • *Participant tracking techniques* to document changes in residential status • *Incentives such as* small gifts timed to baby's milestones • *Consistent communication through* quarterly newsletters physically and electronically mailed to participants • *Participant appreciation *via semi-annual events to maintain contact • *Sustained strong clinical partnerships* with community-based health care providers with high proportions of medically under-served and research underserved population
Begum 2012 [58]	Clinic settings (Physician offices)	Pregnant people were recruited in their first trimester in physician offices	Jun 2009-Jun 2010	**Engagement & Recruitment:** • *Barriers to engaging with other groups* where participants are relatively homogenous and lack of diversity by ethnicity, maternal age, education, marital status, or family income **Retention:** Not reported	**Engagement & Recruitment:** Not reported **Retention:** Not reported
Chasan-Taber 2009 [59]	Clinical settings (Ambulatory obstetrical practices)	Prenatal care patients were recruited at their first prenatal care visit by bilingual recruiters	Jan 2006-Aug 2008	**Engagement & Recruitment:** • *Cancellation of appointments* impact recruitment in clinic settings • *Inequities in engaging with people with sociodemographic representativeness* in study population with participants being young and unmarried, consistent with prior studies Hispanic people encountering social inequities and barriers from very young childbearing ages • *Logistical challenges* when recruiters miss potential participants and clinic staff that are already overburdened **Retention:** • *Low attendance to pre-natal visits* be due to personal and **child sickness, domestic tasks, unanticipated employment opportunities**, and **partner restrictions**	**Engagement & Recruitment:** • *Cooperation from clinic staff* before the onset of the study with regular meetings to explain the purpose of the study and receive feedback on study protocols • *Bilingual recruiters* in the clinic focused on recruiting patients at the time of their regularly scheduled prenatal care visit • *Recruiter training* involving being female recruiters and bilingual to offer simple, clear information with flexibility and accommodation to people • *Pre-screening process* to increase efficiency where recruiters used a limited number of available demographic (i.e., date of birth) and medical characteristics (i.e., date of last menstrual period) based on a daily roster of scheduled patients to generate a list of potential participants • *Culturally tailored materials* such as eligibility screening forms, informed consent forms, HIPAA forms, questionnaires, posters, fliers, and patient handouts all translated into Spanish • *Participant compensation* including variety of items (e.g., small teddy bear, baby t-shirt, hat and bib, gift certificates) with study logo and contact information • Administer questionnaire in Spanish or English to eliminate potential language or literacy barriers **Retention:** • *Flexibility in recruitment* by retaining contact information to reach participants if the interview is interrupted • *Voucher (cafeteria)* to complete the interview • *Monitoring recruitment goals* through a data management system to track information about study recruitment • Reduce participation burden by shortening length of questionnaire • *Shorten length of interview* by collecting medical and obstetric history from the patient's medical record
Ernst 2015 [60]	Clinical settings and hospitals	People were recruited during the last trimester of pregnancy, during prenatal care visits at primary care by a gynecologist or in hospital by the attending obstetricians or project staff	Jan 2012-Mar 2013	**Engagement & Recruitment:** • *Time consuming* to establish recruitment and other study procedures within the hospital settings • *Incentives only important to a limited extent* e.g., those with low socio-economic status (SES) • ***Study language limitations*** resulting in only involving people whose knowledge of the Ger- man language was sufficient to understand study materials and details of participation • *Variety of survey instruments and administration techniques* to reach different populations • *Collaboration with multiple institutions*, e.g., maternity units, primary care gynecologists, community services or other services for pregnant people **Retention:** • *Need to* examine further recruitment and (retention) strategies in the **social context (e.g., community-based recruitment**)	**Engagement & Recruitment:** • *Early communication about study* and guideline for enrollment to inform people by primary care gynecologists, hospital staff (midwives, physicians) or by a study nurse • *Direct contact* with expecting people & couples in the context of antenatal care (i.e., midwife consultations) by project members • *Engagement and recruitment by motivated gynecologist, nurse, recruiter* increase likelihood of people taking part in a birth cohort **Retention:** Not reported
Faro 2021 [61]	Clinic and community settings	People were recruited during pregnancy	NA	**Engagement & Recruitment:** • *Challenges in recruiting racially and ethnically diverse study participants* • *Burden of* biospecimen collection and data collection tools **Retention:** • *High participant burden* and stress of coordinating prenatal care combined with anxiety about birth event, changes in lifestyle and family expectations • *Social and family structure potentially a challenge for people to participate*	**Engagement & Recruitment:** • *Recruitment in multiple languages* to address potential **literacy barriers** • *Provide incentives* for participation • *Record and keep track* of reasons for non-enrollment • *Partnerships with organizations* that work within the community of interest to recruit minority populations **Retention:** Not reported
Garcia-Blanco 2018 [62]	Clinical settings	People were recruited during the third trimester of pregnancy	Jan 2015-Dec 2015	**Engagement & Recruitment:** • *Establish clinical-academic-community partnerships* at all stages of the study **Retention:** Not reported	**Engagement & Recruitment:** • *Clinical-academic-community partnerships* can improve research efficiency and accelerate the recruitment and data collection phases of a study • *Incentives* such as public library gift certificates and grocery gift certificates for completed questionnaires • *Convenience mailing* with postage paid envelopes to minimize time and costs **Retention:** • *Consistent communication* such as phone calls for outstanding questionnaires
Gracie 2010 [63]	Clinical settings	People who receive prenatal viral serology testing were recruited through a partnership with clinical laboratory services	Sep 2009- Dec 2010	**Engagement & Recruitment:** Not reported **Retention:** • Not reported	**Engagement & Recruitment:** • Not reported **Retention:** • *Consistent communication* such as reminder phone calls for outstanding questionnaires • *Incentives,* such as public library gift certificates and grocery gift certificates for completed questionnaires
Hertz-Picciotto 2010 [64]	Community settings	Telephone interviews, internet-based surveys, and home-based monitoring techniques were used to recruit families (one parent and one child) and older individuals (age 55 +)	Jul 2005-Jan 2007	**Engagement & Recruitment:** • *Participation motivation and passive refusal* impacted by lack of time and interest • *Lack of trust* impacted sampling from **diverse communities, low SES, who are disproportionately impacted by toxins** **Retention:** • *Cohort length and time burden* are important determinants of participation and retention • *Participant burden* particularly for **families with young children**	**Engagement & Recruitment:** • *Oversampling of hard-to-reach populations* can be effective in leading to a more diverse sample **Retention:** • *Reduce participant burden time* by conducting home visits • *Deploy the same study staff* to build and maintain trust
Hertz-Picciotto 2018 [65]	Community settings (State-level agency that coordinates services for persons with developmental disabilities)	Families on the state-level agency list with a child with Autism Spectrum Disorder were mailed a letter notifying them about the MARBLES study, before or during a pregnancy, and once the woman became pregnant, they were invited to enroll	July 2005-Jan 2007	**Engagement & Recruitment:** Not reported **Retention:** Not reported	**Engagement & Recruitment:** Not reported **Retention:** Not reported
Kawamoto 2014 [66]	Clinical settings and Community (local government offices)	People were recruited at their first prenatal examination at cooperating health care providers (obstetric facilities) and through community-based recruitment at local government offices	Jan 2011-Mar 2014	**Engagement & Recruitment:** Not reported **Retention:** Not reported	**Engagement & Recruitment:** • *Consistent communication* with participants via telephone calls to continue engagement **Retention:** Not reported
Lara-Cinisomo 2016 [67]	Clinical settings	People were recruited during prenatal visit and community centres	Jul 2013-Apr 2014	**Engagement & Recruitment:** • *Issues of confidentiality* can be especially important to vulnerable populations, such as pregnant and Spanish-speaking people • *Study setting (and traveling to study site)* an important indicator of Latina people likely to participate and enrol in the study • *Conduct community-based* needs assessments to determine research areas of interest, mental health needs among perinatal native and U.S.-born Latin people to engage and recruit • *Fear of being rude* to refuse participation **Retention:** • *Geography as a barrier* when many live outside of the study catchment area and must travel long distance to receive prenatal care • *Culturally appropriate methods* to ensure participation and retention	**Engagement & Recruitment:** • *Bilingual investigators and recruiting staff* to enrol participants • *Consistent communication* by providing a cell phone number for participants to inquire about the study • *Availability and accessibility of PI* when greeting people at in-person and phone interviews to build trust • *Establish trust early* in the study (at enrollment) by providing participants brief reports and preliminary results from the study • *Build trust and relationships* by assuring participants that information will not be shared with government agencies **Retention:** • *Consistent communication* such as follow-up and confirmations letters at all points to thank participants and provide reminders of future appointments • ***Translation of material*** in participants’ preferred languages (English and Spanish)
Loubet 2016 [68]	Online	Pregnant people were enrolled through a web-based platform	Nov 2014-Apr 2015	**Engagement & Recruitment:** • *Non-representativeness* of study sample • *Challenges with "volunteer effect"*—those who choose to volunteer for studies may differ in lifestyle and health from those who decline) **Retention:** • *Longer follow-up periods have higher chances for dropout*	**Engagement & Recruitment:** • *Web-based survey* cover a wider geographical distribution of participants and achieve a high active participation rate **Retention:** Not reported
Loxton 2015 [38]	Online	People were recruited for the longitudinal online survey. Promotions were made via social media (Facebook), promotion, by referral, web activities, and via traditional media outlets	Oct 2012-Dec 2013	**Engagement & Recruitment:** • Referrals are most likely and social media less likely to support engagement and recruitment • *Costs of advertising* will need to be considered • *Challenges in recruiting hard-to-reach populations* via social media channels **Retention:** Not reported	**Engagement & Recruitment:** • *Social media most successful* to recruit young people and achieve demographic diversity **Retention:** Not reported
McDonald 2013 [86]	Clinical and Community settings	People were recruited from health care offices, communities, and through Calgary Laboratory Services	May 2008-Dec 2010	**Engagement & Recruitment:** • *Non-response bias throughout the study* need to be understood through social contexts, inequities and wellbeing **Retention:** Not reported	**Engagement & Recruitment:** • *Incentives* such as library and grocery store gift cards. In order to keep participants engaged and updated • *Milestone communication* such as congratulation cards baby’s birthdays • *Study newsletters and updates* on preliminary results **Retention:** Not reported
Manca 2013 [69]	Clinical settings (Physician offices)	People were recruited face-to-face in physician offices, distributing posters and pamphlets, word-of-mouth, media, and the Internet	May 2009-Nov 2010	**Engagement & Recruitment:** • *Geography and study setting* difficult to reach site by transit • *High SES participants* more likely to participate in the APrON **Retention:** Not reported	**Engagement & Recruitment:** • *Variety of recruitment strategies:* 1) Face-to-face recruitment through physician offices and high-volume maternity clinics; 2) Honorarium for clinic staff to discuss the study with patients and attain contact information; 3) Advertising (posters and flyers in public spaces that pregnant people were likely to visit; 4) Word-of-mouth from health care providers, staff, friends and family of participants; 5) Media outlets with the greatest readership and audiences, as well as those related to pregnancy and nutrition; 6) Internet • *Face-to-face strategies* in physician offices were the most successful in recruiting people • *Collaboration with relevant organizations* (Doula Association, Association for Safe Alternatives in Childbirth) *and professionals* (midwives and naturopath clinics), *centres* for pregnant teens, *programs* to support low-income pregnant people, *community perinatal programs* and the *provincial after hours medical help line* enhanced recruitment strategies **Retention:** Not reported
Morrens 2017 [81]	Clinical settings (Maternity hospitals as primary sampling units (PSU))	Pregnant people were informed midwives and invited to participate. Study nurses provided potential participants with detailed information about the study protocol and recruited participants	Nov 2013-Nov 2014	**Engagement & Recruitment:** • *Overcoming an intuitive ‘no’* by eliminating fear or perceived danger of being asked to participate • ***Mistrust*** by provided information about data use • *Identify perceived benefit* by reassuring participants that study participation would not involve risks • *Frame the utility and benefits of study participation* more in terms of ‘personal profit’ by offering more personalized information on how to avoid or protect themselves against exposure to environmentally hazardous chemicals • *Avoiding research triage* as socially vulnerable people often leave the hospital after delivery and are more difficult to reach for the purpose of research studies **Retention:** Not reported	**Engagement & Recruitment:** • *Community engagement and consultation* to modify study procedures and ask questions (how do socially vulnerable pregnant people experience environmental health risks); and understand participation barriers for human biomonitoring research (which study procedures may cause barriers for socially vulnerable people?), identify opportunities to increase participation (how can we motivate pregnant people?) • *Network with community organizations and local professionals* to achieve broader publicity and endorsement of the study • *Stimulate word-of-mouth promotion* within communities in the catchment areas • *Implement a personal buddy system* for participants to build *trust and personal relationships with potential participants* • *Invest in information transfer* to eligible candidates about the study process in the pre-parturition period **Retention:** Not reported
Morton 2014 [70]	Clinical settings	Lead maternal carers recruited pregnant people into the study at routine appointments	Apr-25 2009-Mar 25, 2010	**Engagement & Recruitment:** • *Need to address challenge of recruiting people from diverse communities an ongoing issue* **Retention:** • *High attrition due to missing contact information for follow-up appointments*	**Engagement & Recruitment:** • *Consultation and collaboration strategies* with relevant health care and community organizations • *Communicating and working with diverse population* to enable antenatal enrollment • *Promote awareness* through indirect and direct contact with prospective participants and communities • *Advertising and media locations in areas and* languages most frequently spoken in the study region **Retention:** • *Up to date contact details* to reduce attrition • *Establish good rapport between interviewers and participants to build trust* • *Translation and relationship building* to match interviewers with participants based on ethnicity, language, and availability (preferred time and location) • *Flexibility of scheduling* to minimize inconvenience and burden for participants • *Satisfaction with the study and continued engagement* • *Address attrition by reducing dissatisfaction* with the study or its methods • *Auditing is essential* to manage in-person antenatal interviews by an external research company for assuring accurate data acquisition • *Continuing engagement strategies* and regular contact with the participants to provide access to resources, initial findings, information regarding privacy and confidentiality, and electronic copies of the participant newsletters
Postma 2016 [71]	Community settings	Subjects were recruited via household-based door-to-door within randomly selected, pre-determined geographical areas	2010–2011	**Engagement & Recruitment:** • Need variety of approaches to promoting outreach and engagement strategies across different media outlets (i.e., English and Spanish) • Lack of clarity on which certain strategies led more credibility than other strategies **Retention:** Not reported	**Engagement & Recruitment:** • *Application of cultural responsiveness theory* helpful to structure outreach and engagement, including: • assembling a culturally competent team; • partnering with community organizations; • creating a personalized marketing and media campaign • *Hiring locally* so the staff hired reflected the county, and represented a cross-section of age, gender, ethnicity, and academic backgrounds • *Staff serving as* “bridge” between researchers, community partners and community members at-large **Retention:** Not reported
Quante 2012 [72]	Clinic and community settings	Subjects were recruited at university hospitals, local clinics, public health centres, kindergartens, schools and partner study centres	Jul 2011-Jul 2014	**Engagement & Recruitment:** Not reported **Retention:** Not reported	**Engagement & Recruitment:** • *Consistent communication* with participants throughout the stages of the study • *Study promotion* through presentations, forums, fliers/posters, press releases and TV ads **Retention:** • *Build relationships* with participants involving invitations and opportunities to participate in parties and sport events for children
Richiardi 2007 [73]	Online (Internet-based)	Registered people were asked to complete three Internet-based questionnaires during pregnancy and 6 and 18 months after delivery and consent for passive follow-up of the child and the mother, using linkage with records of health-related databases, such as the Hospital Discharge Registry and cancer registries	July 2005-Dec 2006	**Engagement & Recruitment:** • *Selection bias* with participants being different from the general population of pregnant people both due to access to the Internet and were self-selected volunteers (highly educated, high SES and older) • *Internet-based recruitment poses* concerns about privacy **Retention:** • *Lack of contact with the participants*, creating challenges for collection of biological samples	**Engagement & Recruitment:** • *Multiple promotion strategies* such as posters at the main hospitals of the city; leaflets enclosed with the results of laboratory tests and ultrasounds carried out for the prenatal screening; leaflets distributed at the pre-delivery classes after giving a brief overview of the study • *Strategies for Internet-based recruitment* is a more long-term and sustainable approach over several years including period of recruitment and follow-up • *“Anonymity” of an Internet-based questionnaire may yield higher response rates*, and more accurate responses, for questions of a personal nature **Retention:** • *Internet-based recruitment and engagement* facilitates communication with participants and reduces loss to follow up
Smith 2021 [74]	Community settings	People were recruited via traditional, social media and broadcast email outreach	Apr 2018-Jan 2020	**Engagement & Recruitment:** • *Social media as an approach alone may not be entirely effective as recruiting hard-to-reach people* who are less likely to enroll and participate in the study • *Lack of interest or distrust in scientific research* • *Disapproval from family and friend* • *Time/participant burden* **Retention:** Not reported	**Engagement & Recruitment:** • Social media has the potential to reach hard to recruit populations such as people experiencing pregnancy-related depression, but in combination with other traditional strategies and efforts to *build trust, relationship, and acknowledge institutional royalty* may be more effective • *Perceived personal benefit* may motivate people to participate (i.e., improved pregnancy outcome, health education, and improvements to their own health • *Social media recruitment requires minimal efforts and cuts time burden* **Retention:** Not reported
Spallek 2020 [75]	Clinical settings	People were recruited by gynecologists and midwifes as well as via ads and flyers	2016–2018	**Engagement & Recruitment:** • *Lower response rates* impacted by participants being from migrant backgrounds and lower educated families **Retention:** Not reported	**Engagement & Recruitment:** • *Culturally sensitive recruitment and bilingual study materials and study nurses* to recruit and follow-up as many pregnant people as possible independent from their social or cultural background **Retention:** Not reported
von Ruesten 2014 [76]	Clinical settings	Subjects were sent mail invitations after a routine ultrasound examination at local hospital	1999–2008	**Engagement & Recruitment:** • *Ensuring representativeness* of all pregnant people and fathers in the region **Retention:** Not reported	**Engagement & Recruitment:** • Not reported **Retention:** Not reported
Walker 2011 [77]	Clinical settings (Antenatal)	Recruitment was performed by experienced obstetrical research personnel	Oct 2002-Apr 2009	**Engagement & Recruitment:** Not reported **Retention:** Not reported	**Engagement & Recruitment:** Not reported **Retention:** Not reported
Webster 2012 [78]	Clinical settings (Hospital)	People were recruited by clinic staff and various promotions strategies (i.e., advertising mediums, website, media, online and recruitment emails, as well as a study booth at baby “trade shows”)	Oct 2006-Feb 2008	**Engagement & Recruitment:** • *Direct contact *via* physicians and midwives*—expensive and unsuccessful, due to late start in implementing this strategy, the difficulty of communicating directly with clinicians (e.g., by phone or email), the high volume of research requests, concern about the study topic, and the low numbers of eligible people visiting a clinic on any given day • *Challenge with recruiting people from diverse groups, younger, ethnically diverse* (predominantly Caucasian), less affluent and educated **Retention:** Not reported	**Engagement & Recruitment:** • *Involving prominent members* of the clinical community (senior midwives, family physicians and obstetricians) from the beginning of the study to facilitate recruitment • *Word-of-mouth as the most cost-effective recruitment* method (i.e., forwarding of messaging to friends and family, referral from another study, and direct recruitment emails) • *Availability of posters and flyers where people frequently visit* (participating hospitals, at family practice clinics, midwifery clinics, medical testing laboratories, an ultrasound clinic, maternity and newborn retail outlets, libraries, community centres, yoga studios, grocery and natural foods stores, coffee shops, community message boards, and at select naturopathic, chiropractic, massage therapy, and physiotherapy clinics) • *Online recruitment methods* effective when there is comprehensive study information • *Recruitment booths* (CHirP study recruitment booth) at baby and pregnancy trade shows, family physician, midwifery and doula conferences, an outdoor summer yoga event and at summer farmers’ markets • *Raise awareness and knowledge sharing/exchange about recruitment strategies with care providers *via powerpoint presentations to obstetricians, family physicians, midwives, nurses and other hospital staff during research rounds and staff meetings at the three participating hospitals • *Hiring* e*nthusiastic and knowledgeable study staff* to build relationships and trust • *Incentives* such as hospital parking passes or bus tickets, a baby T-shirt, and their personal results at the end of the study (chemical levels measured in their blood and homes, shared at least 1 year after all babies had been born), offering genuine thanks via small gifts and cards, and offering to share personal results with participants helped us to recruit 152 people ≤ 15 weeks gestation within 17 months; and follow up visits available when needed **Retention:** Not reported
Zook 2010 [79]	Clinical settings	People were recruited by clinic staff at four sites (Baltimore, Boston, New York, and St. Louis)	Feb 2005-Mar 2007	**Engagement & Recruitment: Retention:** Not reported **Retention: Retention:** Not reported	**Engagement & Recruitment:** • *Keeping contact* to help locate participants who move or change phone numbers by asking mothers at the initial study visit, to provide the names, telephone numbers, and addresses of up to three family members, neighbors, or friends who would be willing to help the staff communicate with the mother • *Establish and build relationships* with clinic personnel at recruitment sites to increase study awareness • *Hire culturally competent and culturally sensitive staff* with strong interpersonal skills • *Identify supporting clinics* in the catchment area • *Promotions and presentations* at child health community programs and in clinic areas **Retention:** • *Monitor staff assignments and effectiveness of recruiting strategies* with assignment delegation • *Establish minimum number of required* call attempts for completion of study calls/visits • *Collect name/contact information for alternate contacts* at the initial interview • *Allow for after-hours staffing* to complete calls • *Purchase site cell phones* to use for calling participants who are difficult to reach and who may not answer a hospital number • *Tracking/monitoring reports* to chart the study progress and identify problem areas • *Mail appointment letters* and follow-up letters to participants who are difficult to reach, monthly postcards as reminders of study events or check-in requests, and greeting cards

#### Participant burden

Time required to participate in study visits was a recurrent theme in the reviewed literature and highlighted by nine studies [37, 55, 57, 60, 61, 64, 70, 74, 76]. In addition to needing to seek time off from work or care-giving responsibilities, participants may be asked to travel for interviews and incur transportation costs. These demands combined with stressors related to caregiver burden (e.g., child care and elder care), job constraints, and living in socioeconomically deprived neighborhoods may also deter participation [56, 59]. Retention may be similarly affected by high participant burden.

#### Socioeconomic inequities

As noted in six studies, marginalized populations experience greater challenges in regularly seeking and accessing health care due to language and literacy abilities [56, 59–61, 67, 70], level of education attainment, living in socio-economically deprived households, or experiences of stigma and exclusion associated with racism [56, 58, 59, 80]. These challenges in health care access transcend to study participation. Antenatal clinics are the most common settings for pregnancy cohort study recruitment and engagement; however, individuals who do not have antenatal care, due to socioeconomic status related barriers, will be excluded from recruitment efforts and, as a result, not invited to participate in the study [57, 76, 81]. Individuals with language barriers, lack of digital literacy, or limited internet access are also less likely to be recruited into studies that use online tools for engagement and recruitment [38, 68, 73, 82]. Although online methods can facilitate recruitment from a wider geographic range than in-person recruitment methods, the resulting study population may comprise primarily educated participants of higher socioeconomic status [82].

#### Lack of trust in science and research

Negative experiences with health care services or research, misinterpreted stories, or historical disenfranchisement may all contribute to a lack of trust in science and research. These negative experiences and resulting lack of trust may be exacerbated in individuals and populations who have been subject to disproportionate exposure, injustice or discrimination [22, 25, 83]. Hertz-Picciotto et al*.* reported that people of low socioeconomic status and those who experience disproportionately high exposure to environmental chemicals may be less likely to trust science and the research process [55]. Authors of three studies noted that individuals from marginalized communities may also be hesitant to share personal or biospecimen data with governmental agencies, or any form of authority [67, 74, 81]. This hesitancy may be rooted in concerns regarding confidentiality, data sharing, and privacy [55].

#### Lack of cultural relevance

Recruitment strategies in pregnancy cohort studies that ignore cultural beliefs and norms impede participation of racial or marginalized communities. Questionnaires requesting personal details about the family and home environment may appear invasive or even offensive [31]. Additionally, participants may be hesitant, for cultural or religious reasons, to provide biospecimen samples. For example, Morrens et al*.* report that some Muslim women may view biospecimen samples, such as the placenta or cord blood, as sacred [81]. In contrast to the Western biomedical view of biospecimen samples as either medical waste or a donation to research, individuals who follow certain cultural or religious traditions may assign cultural meanings and representations to biological samples [61]. Barriers related to the lack of cultural relevance were more commonly reported in the initial stages of recruitment when individuals are first presented with research topics and requirements for participation than later stages of the research [59, 67, 75].

### Strategies to facilitate engagement, recruitment, and retention into pregnancy cohort studies

From the reviewed literature, we identified the following primary strategies to facilitate engagement, recruitment, and retention: *minimize participant burden, address social inequities, build trust in science and research,* and *consider cultural relevance* (Table 3). As with the barriers, these strategies were mapped onto the socioecological model of health (Fig. 2) and the study design timeline (Fig. 3).

#### Minimize participant burden

Three studies reported strategies to reduce participant burden including shortening interview questionnaires, reducing time commitment, conducting home visits, and providing flexibility in scheduling and cancelling interviews [55, 59, 61].

#### Address social inequities

To mitigate equity-related barriers (e.g., literacy and language), researchers have engaged with the local community and developed study design and communication materials tailored to the local community context. Eight studies reported that multiple strategies are needed to ensure that potential studies are planned at the outset to be inclusive, adaptable, equitable, culturally relevant, and responsive as well as designed to meet the needs, capacity, and interests of participants [55, 56, 59, 64, 74, 75, 80, 81]. Other specific strategies to address social inequities are outlined below.

Early and ongoing consultation and engagement with local community members and leaders, service providers, and community organizations have been highlighted as an important strategy by eight research teams [57, 60–62, 69, 70, 81, 84]. Authors of the Alberta Pregnancy Outcomes and Nutrition (APrON) study reported that collaboration with relevant organizations and professionals (centres for pregnant teens, programs to support low-income pregnant people, community perinatal programs, and the provincial after hours medical help line) enhanced recruitment strategies, particularly for hard-to-reach individuals [69]. Community engagement and consultation can inform study procedures by asking critical questions (*“how do socially marginalized pregnant people experience environmental health risks”)*, understanding barriers to participating in human biomonitoring research *(“which study procedures may cause barriers for socially marginalized people”)*, and identifying opportunities to increase participation *(“how can we motivate pregnant people to participate?”)* [81]. Forming strong partnerships between clinical and community-based care providers who work with underserved and disadvantaged communities may also be an effective strategy for engagement, recruitment, and retention within those populations [57, 61, 62]. Engagement and recruitment by motivated health care providers (e.g., gynecologist, nurse), in collaboration with researchers, increase the likelihood of pregnancy cohort participation particularly if the relationships between care providers and participants are well-established and based on trust [61, 84].

Recognizing that research projects aiming to include marginalized populations require sustained communication, authors of multiple studies identified both strategies and barriers relevant to effective communication. Fourteen studies reported that sustained communication strategies (i.e., via quarterly or semi-annual newsletters) to express participant appreciation, share study updates and preliminary results, and celebrate babies’ milestones are effective means of maintaining participant retention in longitudinal research [37, 38, 58, 62, 66, 68, 69, 71–73, 79, 81, 85, 86]. Despite the ease of online communication, strategies using this medium need to consider potential barriers resulting from lack of internet access, electronic devices, and digital literacy. Internet-based communication, such as sending appointment reminders and providing study updates, may aid with participant retention [73].

Six studies reported that providing compensation and incentives can help maintain participation throughout the duration of longitudinal research recruitment and follow-up [57, 61–63, 78, 86]. Examples of compensation and incentives include grocery store gift cards [62, 78, 86], baby T-shirts, and small gifts timed to baby's milestones [87]. Covering the costs incurred by participation (e.g., transportation, parking) is another strategy for minimizing social inequity barriers [78, 79]. Thus, although study compensation and incentives cannot remove social inequities, these strategies may help mitigate some of the barriers imposed by participation.

#### Building trust in science and research

Building trust between the research team and participants is instrumental to successful recruitment and retention [64, 67, 74] and, as noted by six studies, may be accomplished, in part, by providing information on how data will be collected, handled, stored, and reported [37, 38, 55, 59, 64, 74]. An additional reported strategy to build trust in the research process was a ‘buddy system’[81]. Buddies may be third-party individuals with similar ethnic backgrounds who are hired by the research team and can foster meaningful social connections with the community being engaged and recruited. Morrens et al., used this approach to support women at all stages of the research to provide opportunities to ask questions, discuss the study with family members, and facilitate informed decisions [81]. A ‘bridge person’ is another strategy to address mistrust, particularly in research that involves engaging with racialized and marginalized communities [42, 55, 71, 78, 79]. A ‘bridge person’ may be a same-culture researcher, a (trained) community member or leader, or staff from a community-based organization [44]*.*

#### Consider cultural relevance

Tailoring study design materials to participants’ language, cultural norms, and beliefs is integral to reducing language and literacy related barriers and enhancing cultural relevance, as noted by five studies [59, 64, 65, 67, 81]. Translating study materials (e.g., eligibility screening forms, informed consent forms, questionnaires, posters, fliers, and patient handouts) [41] and hiring bilingual staff representative of the local culture are demonstrated strategies for enhancing recruitment and engagement [59, 75, 79]. As an example, Postma et al. aimed to address social inequities in a pregnancy cohort study of Hispanic and non-Hispanic participants by operationalizing cultural responsiveness theory. Specifically, they assembled a culturally competent team, established partnerships with community organizations, and created a tailored and personalized marketing and promotion campaign [62].

Hiring bilingual staff representative of local culture is an effective sequelae of community engagement. Involvement of local staff who are representative of the target population, culturally competent, sensitive, and have strong interpersonal skills benefits participant engagement [70, 75]. Specifically, staff may share their expert knowledge of community connections, form meaningful connections among researchers, staff, community partners, and community members at large [79], as well as build trust and foster relationships between the research team and participants [38, 55]. Local staff may also be able to identify and facilitate support networks for participants; this approach has been shown to enhance participation [55, 71, 78].

## Discussion

This scoping review identified barriers and strategies to improve engagement, recruitment, and retention of individuals from marginalized communities into pregnancy cohort studies. Participant burden, pre-existing social inequities, lack of trust in research, and lack of cultural relevance were the primary identified barriers. Corresponding strategies to bolster participation are rooted in culturally relevant and responsive community-based consultation and engagement that begin in the planning stages and continue throughout the study. Implementing these strategies requires researchers to identify community members and partners early in the study planning process to allow sufficient time to build partnerships and trust. As summarized in Fig. 3, our review identified practical strategies that can be adopted at different stages of engagement, recruitment, and retention to achieve meaningful participation through all stages of the study. Consistent with our interpretation of the literature, Snow et al., in their review of engaging marginalized populations in health services research, concluded that a ‘one-size-fits-all’ approach is insufficient for engaging with marginalized populations to address barriers to participation [88].

The orientation of our review was informed by an interdisciplinary consultation process with knowledge users [53, 54]. The PEHE-MIREC consultative group voiced strong support for the need to find better ways to ensure inclusion and representation of marginalized communities in future Canadian cohorts and offered guidance to support the framing and analysis of the present review. Notably, the consultative process helped to shape our attentiveness to the contextually-driven and dynamic factors that lead to disproportionate barriers to research participation at individual and community levels, as reflected in our use of the socioecological model to frame our findings. Similarly, the depth of practical experience represented within the consultation group helped attune our thematic observations regarding research recruitment and retention strategies that can build trust, increase cultural relevancy, reduce socio-economic barriers and participant burden, and ensure reciprocity.

Mapping the barriers onto the socioecological model of health (Fig. 2) contextualized how the barriers are shaped by prospective research participants’ individual, interpersonal, institutional, community, and policy environments. As well, this model demonstrates how corresponding successful strategies must undertake a multi-pronged approach. For example, researchers have the capacity, and indeed the responsibility, to build trust (interpersonal), reduce participant burden (interpersonal), tailor recruitment strategies (institutional), engage and consult with the community to identify and address community needs (community), and attend to the potential for research findings to inform health-protective measures (policy). We did not identify any specific studies that addressed barriers at the policy level; however, Beasley et al*.* recommend prioritizing measurement of the research impact for the community studied rather than the impact of the research for the researcher [41]. Shifting the focus of impact assessment from researcher achievement towards research program sustainability and lasting value to the community may help facilitate policy level changes relevant to funding structures and calls for research that addresses the needs of marginalized communities. If policy impact is the end goal, participants need to be informed about the policy implications of the research and the individual value of their engagement in the research. In other words, researchers need to be prepared to meaningfully provide answers to questions such as “*why should I participate*” and “*how will this benefit me and my child’s health*.” [55] These messages were also identified in the stakeholder meetings conducted in parallel with this review.

In addition to desired impacts of research on policy, some policy decisions may impede individual level engagement and participation in research [89]. Moreover, contemporary lack of trust in science and research may have roots in historical policies that contributed to structural racism, trauma, and colonial oppression. Although identifying these potential macro-level sources of distrust was not feasible within our review, we acknowledge the need to consider the historical and contemporary local context when planning future studies. Given our focus on informing future Canadian biomonitoring research, it will be important to consider how barriers related to indigeneity, rurality, and race may deter and constrain participation of community members in environmental health and biomonitoring research [90–92].

We hypothesize that interventions designed to address barriers at all levels of the socioecological model are more likely to enhance engagement, recruitment, and retention of individuals than efforts targeted at a subset of levels; however, the existing evidence base does not provide the data to make this comparison. There was no identified study that implemented strategies that corresponded to all or most levels of the socioecological model that could be appropriately compared to – in terms of retention rates – studies that considered a subset of strategies.

We identified multiple knowledge gaps in the reviewed evidence that are important to consider for conceptualization of future pregnancy cohort studies. *First*, no identified studies discussed barriers to engaging and recruiting participants prior to pregnancy and retaining them throughout pregnancy and into the postpartum period. Measurement of environmental chemicals in the preconception time period is particularly important for understanding potential reproductive and developmental toxicity [93]. Existing preconception studies have primarily identified participants through fertility clinics [66, 86] or via online surveys [94, 95]. Both of these recruitment strategies have limited effectiveness in reaching marginalized communities and the resulting study populations have limited sociodemographic diversity. *Second,* although a number of reviewed studies included biospecimen collection [63, 67, 76–78], no study discussed potential strategies for enhancing willingness to provide biospecimens or the level of trust needed to provide consent for biospecimen collection. Obtaining robust understanding of environmental chemical concentrations in individuals from marginalized communities relies on availability of biological specimens because human biomonitoring data are considered the gold standard for evaluating the human burden of environmental chemicals [96]. Participants may be reluctant to provide specimens if they lack trust in the research team or if they have unmet concerns about how their data or specimens will be used in future research. While these concerns may be reasonably addressed in some cases with efforts to listen and respond to participant concerns, this issue is largely untouched in the literature. *Third*, few studies focused on engaging men, fathers, or partners in biomonitoring research. Although cohorts in the United States [61] and New Zealand have collected biospecimens from fathers and involve fathers or partners, there is largely a dearth of paternal information in longitudinal pregnancy cohort studies [85]. One of the few preconception studies of couples reported that associations between male partners’ chemical concentrations and diminished fecundity were stronger and more consistent than associations observed in female partners [97]. In addition to the added value of considering paternal exposures in couple-based health outcomes such as fecundity, engaging male partners promotes a holistic approach to reproductive and child health. *Fourth,* no studies attempted to identify engagement, recruitment, and retention strategies for members of the LGBTQS2 + community. Individuals whose gender identity differs from their biological sex at birth may carry a pregnancy [77]; researchers, therefore, need to consider the unique barriers and identify inclusive strategies to recruit transgender, non-binary, and gender-diverse people. *Finally,* a majority of the studies reported on barriers and strategies for recruitment, with limited to no information on retention. We speculate that this gap in knowledge stems from the difficulty in assessing perceptions among participants lost to follow-up. A previous review reported similar findings whereby recruitment strategies were more frequently discussed than those aimed at retention [31]. In light of these gaps, future research focused on the feasibility of recruiting and retaining prospective parents of diverse socioeconomic, educational, ethnic, and gendered backgrounds into biomonitoring pregnancy cohort studies and assessing the potential value of outlined strategies is warranted.

This is the first identified scoping review to focus on barriers to and strategies for improving engagement, recruitment, and retention of marginalized communities in pregnancy cohort studies that collect biological samples to measure environmental chemicals Our findings will be valuable to future biomonitoring within Canada, particularly for forthcoming efforts to make research more inclusive, accessible and without harm for individuals experiencing marginalization. In addition to our rigorous methodology to identify themes in the literature, the strength of our work is enhanced by our consultative PEHE-MIREC process and our use of the socioecological model to map barriers and strategies. Given our focus on higher income regions, our findings are not generalizable to low-income countries. Furthermore, we acknowledge that we assumed a certain degree of homogeneity within marginalized communities and were not able to investigate the impact of overlapping identities or intersectionality.

## Conclusion

Engagement, recruitment, and retention of underrepresented communities in pregnancy cohort studies pose unique challenges and opportunities for researchers. Although there is limited coverage in the literature on strategies to effectively engage people from marginalized communities in environmental health pregnancy cohort studies, this review highlights that applying a health equity and social justice lens and following the ethos of *‘nothing about us, without us’* may help address barriers that exist at the individual, interpersonal, community, institutional, and policy levels. These barriers are likely most pronounced among racialized and socio-economically marginalized communities with deeply rooted inequities. Findings from this review may have important implications for planning future longitudinal pregnancy cohort studies and ensuring that communities who are disproportionately affected by environmental chemical exposures may be better represented in research and considered in policy decisions.

## Supplementary Information

Supplementary Material 1. Supplemental Table 1. Preferred Reporting Items for Systematic reviews and Meta-Analyses extension for Scoping Reviews (PRISMA-ScR) Checklist

## Data Availability

No datasets were generated or analysed during the current study.

## References

[1] Wigle DT, Arbuckle TE, Walker M, Wade MG, Liu S, Krewski D. Environmental hazards: evidence for effects on child health. J Toxicol Environ Health B Crit Rev. 2007;10(1–2):3–39. 18074303 10.1080/10937400601034563

[2] Eaton W. The logic for a conception-to-death cohort study. Ann Epidemiol. 2002;12:445–51. 12377420 10.1016/s1047-2797(01)00314-3

[3] Frank J, Di Ruggiero E, McInnes RR, Kramer M, Gagnon F. Large life-course cohorts for characterizing genetic and environmental contributions: the need for more thoughtful designs. Epidemiology. 2006;17:595–8. 17068411 10.1097/01.ede.0000239725.48908.7d

[4] Canova C, Cantarutti A. Population-Based Birth Cohort Studies in Epidemiology. IJERPH. 2020;17:5276. 32717778 10.3390/ijerph17155276 PMC7432312

[5] Lam J, Lanphear BP, Bellinger D, Axelrad DA, McPartland J, Sutton P, et al. Developmental PBDE exposure and IQ/ADHD in childhood: a systematic review and meta-analysis. Environ Health Perspect. 2017;125(8):086001. 28799918 10.1289/EHP1632 PMC5783655

[6] Rauh VA, Margolis AE. Research Review: Environmental exposures, neurodevelopment, and child mental health - new paradigms for the study of brain and behavioral effects. J Child Psychol Psychiatry. 2016;57(7):775–93. 26987761 10.1111/jcpp.12537 PMC4914412

[7] Lanphear BP. The Impact of Toxins on the Developing Brain. Annu Rev Public Health. 2015;36:211–30. 25581143 10.1146/annurev-publhealth-031912-114413

[8] Office of the Surgeon General (US); Office on Smoking and Health (US). The Health Consequences of Smoking: A Report of the Surgeon General. Atlanta (GA): Centers for Disease Control and Prevention (US); 2004. 20669512

[9] Domagala-Kulawik J. Effects of cigarette smoke on the lung and systemic immunity. J Physiol Pharmacol. 2008;59(Suppl 6):19–34. 19218630

[10] Wigle DT, Arbuckle TE, Turner MC, Bérubé A, Yang Q, Liu S, Krewski D. Epidemiologic evidence of relationships between reproductive and child health outcomes and environmental chemical contaminants. J Toxicol Environ Health B Crit Rev. 2008;11(5–6):373–517. 18470797 10.1080/10937400801921320

[11] Grigg J. Environmental toxins; their impact on children’s health. Arch Dis Child. 2004;89(3):244–50. 10.1136/adc.2002.022202. 14977703 10.1136/adc.2002.022202 PMC1719840

[12] Jarup L. Hazards of heavy metal contamination. Br Med Bull. 2003;68:167–82. 14757716 10.1093/bmb/ldg032

[13] Jarup L, Berglund M, Elinder CG, Nordberg G, Vahter M. Health effects of cadmium exposure – a review of the literature and a risk estimate. Scand J Work Environ Health. 1998;24(Suppl 1):1–51. 9569444

[14] Murata K, Grandjean P, Dakeishi M. Neurophysiological evidence of methylmercury neurotoxicity. Am J Ind Med. 2007;50:765–71. 17450510 10.1002/ajim.20471

[15] Bearer CF. The special and unique vulnerability of children to environmental hazards. Neurotoxicology. 2000;21(6):925–34. 11233762

[16] Koopman-Esseboom C, Weisglas-Kuperus N, de Ridder MA, Van der Paauw CG, Tuinstra LG, Sauer PJ. Effects of polychlorinated biphenyl/dioxin exposure and feeding type on infants’ mental and psychomotor development. Pediatrics. 1996;97(5):700–6. 8628610

[17] Rogan WJ, Chen A. Health risks and benefits of bis(4-chlorophenyl)-1,1,1-trichloroethane (DDT). Lancet. 2005;366:763–73. 16125595 10.1016/S0140-6736(05)67182-6

[18] Weselak M, Arbuckle TE, Foster W. Pesticide exposures and developmental outcomes: the epidemiological evidence. J Toxicol Environ Health B Crit Rev. 2007;10:41–80. 18074304 10.1080/10937400601034571

[19] Boersma ER, Lanting CI. Environmental exposure to polychlorinated biphenyls (PCBs) and dioxins – consequences for longterm neurological and cognitive development of the child lactation. Adv Exp Med Biol. 2000;478:271–87. 11065080

[20] Reuben A, Caspi A, Belsky DW, et al. Association of Childhood Blood Lead Levels With Cognitive Function and Socioeconomic Status at Age 38 Years and With IQ Change and Socioeconomic Mobility Between Childhood and Adulthood. JAMA. 2017;317(12):1244–51. 28350927 10.1001/jama.2017.1712 PMC5490376

[21] Hu CY, Qiao JC, Gui SY, Xu KX, Dzhambov AM, Zhang XJ. Perfluoroalkyl and polyfluoroalkyl substances and hypertensive disorders of pregnancy: A systematic review and meta-analysis. Environ Res. 2023;231(Pt 2):116064. 37178750 10.1016/j.envres.2023.116064

[22] Gochfeld M, Burger J. Disproportionate exposures in environmental justice and other populations: the importance of outliers. Am J Public Health. 2011;101 Suppl 1(Suppl 1):S53–63. 21551384 10.2105/AJPH.2011.300121 PMC3222496

[23] Hoover E, et al. Indigenous peoples of North America: environmental exposures. Environ Health Perspect. 2012;120(12):1645–9. 22899635 10.1289/ehp.1205422 PMC3548285

[24] Schildroth S, Geller RJ, Wesselink AK, Lovett SM, Bethea TN, Claus Henn B, Harmon QE, Taylor KW, Calafat AM, Wegienka G, Gaston SA, Baird DD, Wise LA. Hair product use and urinary biomarker concentrations of non-persistent endocrine disrupting chemicals among reproductive-aged Black women. Chemosphere. 2024;361:142442. 38810806 10.1016/j.chemosphere.2024.142442 PMC11217908

[25] Elamurugan K, Esmaeilisaraji L, Strain J, Ziraldo H, Root A, MacDonald H, et al. Social Inequities Contributing to Gestational Diabetes in Indigenous Populations in Canada: A Scoping Review. Can J Diabetes. 2022;46:628–639.e1. 35779989 10.1016/j.jcjd.2022.05.003

[26] Luo ZC, Liu JM, Fraser WD. Large prospective birth cohort studies on environmental contaminants and child health – Goals, challenges, limitations and needs. Med Hypotheses. 2010;74(2):318–24. 19765909 10.1016/j.mehy.2009.08.044 PMC3035639

[27] Price A, Bryson H, Smith A, Mensah F, Goldfeld S. Processes for engaging and retaining women who are experiencing adversity in longitudinal health services research. BMC Health Serv Res. 2019;19(1):833. 31727073 10.1186/s12913-019-4698-5 PMC6854799

[28] Mapes BM, Foster CS, Kusnoor SV, Epelbaum MI, AuYoung M, Jenkins G, et al. Diversity and inclusion for the *All of Us* research program: A scoping review. PLoS ONE. 2020;15(7):e0234962. 32609747 10.1371/journal.pone.0234962 PMC7329113

[29] Leung BM, McDonald SW, Kaplan BJ, Giesbrecht GF, Tough SC. Comparison of sample characteristics in two pregnancy cohorts: Community-based versus population-based recruitment methods. BMC Med Res Methodol. 2013;13(1):149. 24314150 10.1186/1471-2288-13-149 PMC4029181

[30] Muggli E, Curd H, Nagle C, Forster D, Halliday J. Engaging pregnant women in observational research: A qualitative exploratory study. BMC Pregnancy Childbirth. 2018;18(1):334. 30115019 10.1186/s12884-018-1966-z PMC6097433

[31] Goldstein E, Bakhireva LN, Nervik K, Hagen S, Turnquist A, Zgierska AE, et al. Recruitment and retention of pregnant women in prospective birth cohort studies: A scoping review and content analysis of the literature. Neurotoxicol Teratol. 2021;85:106974. 33766723 10.1016/j.ntt.2021.106974 PMC8137666

[32] Porta M, Gasull M, Puigdomènech E, Rodríguez-Sanz M, Pumarega J, Rebato C, Borrell C. Sociodemographic factors influencing participation in the Barcelona Health Survey study on serum concentrations of persistent organic pollutants. Chemosphere. 2009;76(2):216–25. 19386342 10.1016/j.chemosphere.2009.03.030

[33] Arbuckle TE, Fraser WD, Fisher M, Davis K, Liang CL, Lupien N, et al. Cohort profile: the maternal-infant research on environmental chemicals research platform. Paediatr Perinat Epidemiol. 2013;27(4):415–25. 23772943 10.1111/ppe.12061

[34] Letourneau N, Aghajafari F, Bell RC, Deane AJ, Dewey D, Field C, et al. APrON Study Team. The Alberta Pregnancy Outcomes and Nutrition (APrON) longitudinal study: cohort profile and key findings from the first three years. BMJ Open 2022;12(2):e047503. 10.1136/bmjopen-2020-047503 PMC8823238 35131812

[35] Subbarao P, Anand SS, Becker AB, Befus AD, Brauer M, Brook JR, et al. CHILD Study investigators. The Canadian Healthy Infant Longitudinal Development (CHILD) Study: examining developmental origins of allergy and asthma. Thorax 2015t;70(10):998–1000. 10.1136/thoraxjnl-2015-207246 26069286

[36] Adhikari K, Patten SB, Williamson T, Patel AB, Premji S, Tough S, et al. Does neighborhood socioeconomic status predict the risk of preterm birth? A community-based Canadian cohort study. BMJ Open. 2019;9(2):e025341. 30787092 10.1136/bmjopen-2018-025341 PMC6398791

[37] Bartholomew K, Morton S, Atatoa Carr PE, Bandara DK, Grant CC. Provider engagement and choice in the Lead Maternity Carer System: Evidence from Growing Up in New Zealand. Aust N Z J Obstet Gynaecol. 2015;55:323–30. 26172320 10.1111/ajo.12319

[38] Loxton D, Powers J, Anderson AE, Townsend N, Harris ML, Tuckerman R, Pease S, Mishra G, Byles J. Online and Offline Recruitment of Young Women for a Longitudinal Health Survey: Findings From the Australian Longitudinal Study on Women’s Health 1989–95 Cohort. J Med Internet Res. 2015;17(5):e109. 25940876 10.2196/jmir.4261 PMC4468605

[39] Michikawa T, Nitta H, Nakayama SF, Yamazaki S, Isobe T, Tamura K, Suda E, Ono M, Yonemoto J, Iwai-Shimada M, Kobayashi Y, Suzuki G, Kawamoto T; Japan Environment and Children’s Study Group. Baseline Profile of Participants in the Japan Environment and Children's Study (JECS). J Epidemiol 2018;28(2):99–104. 10.2188/jea.JE20170018 PMC5792233 29093304

[40] Mcleroy K, Bibeau DL, Steckler A, Glanz K. An ecology perspective on health promotion programs article in health education quarterly. Health Educ Q. 1988;15(4):351–77. 3068205 10.1177/109019818801500401

[41] Beasley LO, Ciciolla L, Jespersen JE, et al. Best Practices for Engaging Pregnant and Postpartum Women at Risk of Substance Use in Longitudinal Research Studies: a Qualitative Examination of Participant Preferences. Adv Res Sci. 2020;1:235–46. 33134976 10.1007/s42844-020-00019-1 PMC7592139

[42] Goedhart NS, Pittens CACM, Tončinic S, Zuiderent-Jerak T, Dedding C, Broerse JEW. Engaging citizens living in vulnerable circumstances in research: a narrative review using a systematic search. Res Involv Engagem. 2021;7:59. 34479622 10.1186/s40900-021-00306-w PMC8414765

[43] Daley E, Alio A, Anstey EH, Chandler R, Dyer K, Helmy H. Examining barriers to cervical cancer screening and treatment in Florida through a socio-ecological lens. J Community Health. 2011;36(1):121–31. 20559695 10.1007/s10900-010-9289-7

[44] Salihu H. Socio-ecological model as a framework for overcoming barriers and challenges in randomized control trials in minority and underserved communities. Int J MCH AIDS. 2014;3(1):85–95. PMC4948176 27621990

[45] van der Zande ISE, van der Graaf R, Hooft L, van Delden JJM. Facilitators and barriers to pregnant women’s participation in research: A systematic review. Women Birth. 2018;31(5):350–61. 29373261 10.1016/j.wombi.2017.12.009

[46] Frew PM, Saint-Victor DS, Isaacs MB, Kim S, Swamy GK, Sheffield JS, Edwards KM, Villafana T, Kamagate O, Ault K. Recruitment and retention of pregnant women into clinical research trials: an overview of challenges, facilitators, and best practices. Clin Infect Dis. 2014;59(Suppl 7):S400–7. 25425718 10.1093/cid/ciu726 PMC4303058

[47] Arksey H, O’Malley L. Scoping studies: towards a methodological framework. Int J of Soc Res Method. 2005;8(1):19–32.

[48] Levac D, Colquhoun H, O’Brien K. Scoping studies: advancing the methodology. Implement Sci. 2010;5:69. 20854677 10.1186/1748-5908-5-69 PMC2954944

[49] Tricco AC, Lillie E, Zarin W, O’Brien KK, Colquhoun H, Levac D, et al. PRISMA Extension for Scoping Reviews (PRISMA-ScR): Checklist and Explanation. Ann Intern Med. 2018;169(7):467–73. 30178033 10.7326/M18-0850

[50] Covidence systematic review software, Veritas Health Innovation, Melbourne, Australia. Available at https://www.covidence.org. Accessed 8 Mar 2024

[51] Braun V, Clarke V. Thematic analysis. In: Cooper H, Camic PM, Long DL, Panter D. Rindskopf & Sher KJ(Eds.), APA handbook of research methods in psychology. Research designs: Quantitative, qualitative, neuropsychological, and biological. Am Psychol. 2012;(2):57–71.

[52] Pollock D, Peters MDJ, Khalil H, McInerney P, Alexander L, Tricco AC, Evans C, de Moraes ÉB, Godfrey CM, Pieper D, Saran A, Stern C, Munn Z. Recommendations for the extraction, analysis, and presentation of results in scoping reviews. JBI Evid Synth. 2023;21(3):520–32. 36081365 10.11124/JBIES-22-00123

[53] Pollock D, Alexander L, Munn Z, Peters MDJ, Khalil H, Godfrey CM, McInerney P, Synnot A, Tricco AC. Moving from consultation to co-creation with knowledge users in scoping reviews: guidance from the JBI Scoping Review Methodology Group. JBI Evid Synth. 2022;20(4):969–79. 35477565 10.11124/JBIES-21-00416

[54] University of Ottawa, Department of Geography, Environment, and Geomatics. Prenatal Environmental Health Education Collaboration. https://www.pehe-esep.ca. Accessed 8 Mar 2024.

[55] Ashman AM, Collins CE, Weatherall L, Brown LJ, Rollo ME, Clausen D, et al. A cohort of Indigenous Australian women and their children through pregnancy and beyond: the Gomeroi gaaynggal study. J Dev Orig Health Dis. 2016;7(4):357–68. 27080434 10.1017/S204017441600009X

[56] Bartholomew K, Morton S, Atatoa Carr PE, Bandara DK, Grant CC. Early engagement with a Lead Maternity Carer: Results from Growing Up in New Zealand. Aust N Z J Obstet Gynaecol. 2015;55:227–32. 25898783 10.1111/ajo.12291

[57] Bastain TM, Chavez T, Habre R, Girguis MS, Grubbs B, Toledo-Corral C. Study Design, Protocol and Profile of the Maternal And Developmental Risks from Environmental and Social Stressors (MADRES) Pregnancy Cohort: a Prospective Cohort Study in Predominantly Low-Income Hispanic Women in Urban Los Angeles. BMC Pregnancy Childbirth. 2019;19:189. 31146718 10.1186/s12884-019-2330-7 PMC6543670

[58] Begum F, Colman I, McCargar LJ, Bell RC, et al. Gestational Weight Gain and Early Postpartum Weight Retention in a Prospective Cohort of Alberta Women. J Obstet Gynaecol Can. 2012;34(7):637–47. 22742482 10.1016/s1701-2163(16)35316-6

[59] Chasan-Taber L, Fortner RT, Hastings V, Markenson G. Strategies for recruiting Hispanic women into a prospective cohort study of modifiable risk factors for gestational diabetes mellitus. BMC Pregnancy Childbirth. 2009;9:57. 20003350 10.1186/1471-2393-9-57 PMC2799379

[60] Ernst SA, Günther K, Frambach T, Zeeb H. Prenatal recruitment of participants for a birth cohort study including cord blood collection: results of a feasibility study in Bremen. Germany GMS German Med Sci. 2015;13:1612–3174. 10.3205/000208 PMC4397994 25908931

[61] Faro E, Sauder K, Anders AL, Dunlop AL, Kerver JM, McGrath M, et al. Characteristics of Environmental Influences on Child Health Outcomes (ECHO) Cohorts. Wolters Kiuwer Health. 2021;46:230. 10.1097/NMC.0000000000000725 PMC8225566 33993167

[62] García-Blanco A, Diago V, Hervas D, Ghosn F, Vento M, Cháfer-Pericás C. Anxiety and depressive symptoms, and stress biomarkers in pregnant women after in vitro fertilization: a prospective cohort study. Hum Reprod. 2018;33(7):1237–46. 29796614 10.1093/humrep/dey109

[63] Gracie SK, Lyon AW, Kehler HL, Pennell CE, Dolan SM, McNeil DA, et al. All Our Babies Cohort Study: recruitment of a cohort to predict women at risk of preterm birth through the examination of gene expression profiles and the environment. BMC Pregnancy Childbirth. 2010;10:87. 21192811 10.1186/1471-2393-10-87 PMC3022739

[64] Hertz-Picciotto I, Cassady D, Lee K, Bennett DH, Ritz B, Vogt R. Study of Use of Products and Exposure-Related Behaviors (SUPERB): study design, methods, and demographic characteristics of cohorts. Environ Health. 2010;9:54. 20799988 10.1186/1476-069X-9-54 PMC2940867

[65] Hertz-Picciotto I, Schmidt RJ, Walker CK, Bennett DH, Oliver M, Shedd-Wise KM, et al. A Prospective Study of Environmental Exposures and Early Biomarkers in Autism Spectrum Disorder: Design, Protocols, and Preliminary Data from the MARBLES Study. Environ Health Perspect. 2018;16:11. 10.1289/EHP535 PMC6371714 30465702

[66] Kawamoto T, Nitta H, Murata K. et al. Rationale and study design of the Japan environment and children’s study (JECS). BMC Public Health. 2014;14:25. 10.1186/1471-2458-14-25 PMC3893509 24410977

[67] Lara-Cinisomo S, Plott J, Grewen K, Meltzer-Brody S. The feasibility of recruiting and retaining perinatal Latinas in a biomedical study exploring neuroendocrine function and postpartum depression. J Immigr Minor Health. 2016;18(5):1115–23. 26976007 10.1007/s10903-016-0391-5

[68] Loubet P, Guerrisi C, Turbelin,C. *et al.* First nationwide web-based surveillance system for influenza-like illness in pregnant women: participation and representativeness of the French G-GrippeNet cohort. BMC Public Health 2016;16:253. 10.1186/s12889-016-2899-y PMC4788930 26969654

[69] Manca DP, O’Beirne M, Lightbody T, Johnston DW, Dymianiw DL, Nastalska K, Kaplan BJ. The most effective strategy for recruiting a pregnancy cohort: a tale of two cities. BMC Pregnancy Childbirth. 2013;13:75. 23521869 10.1186/1471-2393-13-75 PMC3614477

[70] Morton SM, Grant CC, Carr PE, Robinson EM, Kinloch JM, Fleming CJ, et al. How do you recruit and retain a Prebirth cohort? Lessons learnt from growing up in New Zealand. Eval Health Prof. 2014;37(4):411–33. 23109469 10.1177/0163278712462717

[71] Postma J, Younglove LR, Brooks K. Hispanic Representation in a Longitudinal Birth Cohort Study. J Health Dispar Res Pract. 2016;9(2):4.

[72] Quante M, Hesse M, Döhnert M, et al. The LIFE child study: a life course approach to disease and health. BMC Public Health. 2012;12:1021. 23181778 10.1186/1471-2458-12-1021 PMC3533937

[73] Richiardi L, Baussano I, Vizzini L, Douwes J, Pearce N, Merletti F; NINFEA cohort. Feasibility of recruiting a birth cohort through the Internet: the experience of the NINFEA cohort. Eur J Epidemiol 2007;22(12):831–7. 10.1007/s10654-007-9194-2 17955333

[74] Smith R, Alvarez C, Crixell S, Lane MA. The Food, Feelings, and Family Study: comparison of the efficacy of traditional methods, social media, and broadcast email to recruit pregnant women to an observational, longitudinal nutrition study. BMC Pregnancy Childbirth. 2021;21:203. 33711946 10.1186/s12884-021-03680-1 PMC7953646

[75] Spallek J, Scholaske L, Kurt M, Lindner-Matthes D, Entringer S. Intergenerational transmission of health disparities among Turkish-origin immigrants in Germany: study protocol of a multi-centric cohort study (BaBi-stress and BaBeK study). BMC Pregnancy Childbirth. 2020;20:158. 32164606 10.1186/s12884-020-2853-y PMC7069210

[76] von Ruesten A, Brantsaeter AL, Haugen M, Meltzer HM, Mehlig K, Winkvist A, Lissner L. Adherence of pregnant women to Nordic dietary guidelines in relation to postpartum weight retention: results from the Norwegian Mother and Child Cohort Study. BMC Public Health. 2014;14:75. 24456804 10.1186/1471-2458-14-75 PMC3908932

[77] Walker MC, Finkelstein SA, Rennicks White R, Shachkina S, Smith GN, Wen SW, Rodger M. The Ottawa and Kingston (OaK) Birth Cohort: development and achievements. J Obstet Gynaecol Can. 2011;11:1124–33. 10.1016/s1701-2163(16)35080-0 22082786

[78] Webster GM, Teschke K, Janssen PA. Recruitment of healthy first-trimester pregnant women: lessons from the Chemicals, Health & Pregnancy study (CHirP). Matern Child Health J. 2012;16(2):430–8. 21210200 10.1007/s10995-010-0739-8

[79] Zook PM, Jordan C, Adams B, Visness CM, Walter M, Chen A, et al. Retention strategies and predictors of attrition in an urban pediatric asthma study. Clin Trials. 2010;7(4):400–10. 20571137 10.1177/1740774510373798 PMC3374495

[80] Bartholomew K, Morton S, Atatoa Carr PE, Bandara DK, Grant CC. Provider engagement and choice in the Lead Maternity Carer System: Evidence from Growing Up in New Zealand. Aust N Z J Obstet Gynaecol. 2015;55:323–30. 26172320 10.1111/ajo.12319

[81] Morrens B, Den Hond E, Schoeters,G. et al. Human biomonitoring from an environmental justice perspective: supporting study participation of women of Turkish and Moroccan descent. Environ Health. 2017;16:48. 10.1186/s12940-017-0260-2 PMC5437637 28526013

[82] Wise LA, Rothman KJ, Mikkelsen EM, Stanford JB, Wesselink AK, McKinnon C, Gruschow SM, Horgan CE, Wiley AS, Hahn KA, Sørensen HT, Hatch EE. Design and Conduct of an Internet-Based Preconception Cohort Study in North America: Pregnancy Study Online. Paediatr Perinat Epidemiol. 2015;29(4):360–71. 26111445 10.1111/ppe.12201 PMC4662659

[83] Cronin M. Anarcha, Betsey, Lucy, and the women whose names were not recorded: The legacy of J Marion Sims. Anaesth Intensive Care. 2020;48(3_suppl):6–13. 33249851 10.1177/0310057X20966606

[84] Korver N, Quee PJ, Boos HBM, Simons CJP, de Haan L, et al. Genetic Risk and Outcome of Psychosis (GROUP), a multi site longitudinal cohort study focused on gene–environment interaction: objectives, sample characteristics, recruitment and assessment methods. Int J Methods Psychiatr Res. 2012;21(3):205–21. 22419500 10.1002/mpr.1352 PMC6878383

[85] Sharp GC, Lawlor DA. Paternal impact on the life course development of obesity and type 2 diabetes in the offspring. Diabetologia. 2019;62(10):1802–10. 31451867 10.1007/s00125-019-4919-9 PMC6731203

[86] McDonald SW, Lyon AW, Benzies KM, et al. The All Our Babies pregnancy cohort: design, methods, and participant characteristics. BMC Pregnancy Childbirth. 2013;13:S2. 23445747 10.1186/1471-2393-13-S1-S2 PMC3561154

[87] Park B, Choi EJ, Ha E, Choi JH, Kim Y, Hong YC, et al. A study on the factors affecting the follow-up participation in birth cohorts. Environ Health Toxicol. 2016;31:e2016023. 28118701 10.5620/eht.e2016023 PMC5198821

[88] Snow ME, Tweedie K, Pederson A. Heard and valued: the development of a model to meaningfully engage marginalized populations in health services planning. BMC Health Serv Res. 2018;18:181. 29544486 10.1186/s12913-018-2969-1 PMC5856315

[89] Willis MD, Hoffman MN, Wang TR, Sabbath EL, Kuriyama AS, Wesselink AK, Wise LA. Evaluating participant engagement in a preconception cohort study in relation to the Dobbs decision. Paediatr Perinat Epidemiol. 2024;38(7):627–34. 38666636 10.1111/ppe.13080 PMC11427157

[90] Nguyen NH, Subhan FB, Williams K, Chan CB. Barriers and Mitigating Strategies to Healthcare Access in Indigenous Communities of Canada: A Narrative Review. Healthcare (Basel). 2020;8(2):112. 32357396 10.3390/healthcare8020112 PMC7349010

[91] Wilson R, Rourke J. Report card on access to rural health care in Canada. Rural Remote Health. 2023;23(1):8108. 36802686 10.22605/RRH8108

[92] Boyer Y. Healing racism in Canadian health care. CMAJ. 2018;189(46):E1408–9. 10.1503/cmaj.171234. 10.1503/cmaj.171234 PMC5698028 29158453

[93] Sun C, Velazquez MA, Fleming TP. Chapter 3: DOHaD and the Periconceptional Period, a Critical Window in Time in *The Epigenome and Development Origins of Health and Disease*. Academic Press 2016; 33–47.

[94] Messerlian C, Williams PL, Ford JB, Chavarro JE, Mínguez-Alarcón L, Dadd R, et al and the EARTH Study Team. The Environment and Reproductive Health (EARTH) Study: A Prospective Preconception Cohort. Hum Reprod Open 2018;2018(2):hoy001. 10.1093/hropen/hoy001 PMC5990043 29888739

[95] Harville EW, Mishra GD, Yeung E, Mumford SL, Schisterman EF, Jukic AM, et al. The Preconception Period analysis of Risks and Exposures Influencing health and Development (PrePARED) consortium. Paediatr Perinat Epidemiol. 2019;33(6):490–502. 31659792 10.1111/ppe.12592 PMC6901022

[96] Sexton K, Needham LL, Pirkle JL. Human Biomonitoring of Environmental Chemicals. Measuring chemicals in human tissues is the “gold standard” for assessing people’s exposure to pollution. Centre for Disease Control and Prevention. Am Sci. 2004;92:38–45.

[97] Buck Louis GM, Barr DB, Kannan K, Chen Z, Kim S, Sundaram R. Paternal exposures to environmental chemicals and time-to-pregnancy: overview of results from the LIFE study. Andrology. 2016;4(4):639–47. 27061873 10.1111/andr.12171 PMC4961554

